# Influence of interdisciplinary frailty screening on perioperative complication rates in elderly ovarian cancer patients: results of a retrospective observational study

**DOI:** 10.1007/s00404-022-06850-4

**Published:** 2022-11-24

**Authors:** Katharina Anic, Jakub Varchola, Mona Wanda Schmidt, Roxana Schwab, Valerie Catherine Linz, Marcus Schmidt, Roland Hardt, Erik Kristoffer Hartmann, Christian Ruckes, Annette Hasenburg, Marco Johannes Battista

**Affiliations:** 1 Department of Gynecology and Obstetrics, University Medical Center of the Johannes Gutenberg University Mainz, Langenbeckstr. 1, 55131 Mainz, Germany; 2 Department of Geriatric Medicine, University Medical Center of the Johannes Gutenberg University, Mainz, Germany; 3 Department of Anesthesiology, University Medical Center of the Johannes Gutenberg University, Mainz, Germany; 4 Interdisciplinary Center Clinical Trials, University Medical Center Mainz, Mainz, Germany

**Keywords:** Frailty, Surgery, Ovarian cancer, Management, Outcome

## Abstract

**Purpose:**

Frailty is a frequent and underdiagnosed multidimensional age-related syndrome, involving decreased physiological performance reserves and marked vulnerability against major stressors. To standardize the preoperative frailty assessment and identify patients at risk of adverse surgical outcomes, commonly used global health assessment tools were evaluated. We aimed to assess three interdisciplinary preoperative screening assessments to investigate the influence of frailty status with in-hospital complications irrespective of surgical complexity and radicality in older women with ovarian cancer (OC).

**Methods:**

Preoperative frailty status was examined by the G8 geriatric screening tool (G8 Score-geriatric screening), Eastern Cooperative Oncology Group performance status (ECOG PS-oncological screening), and American Society of Anesthesiologists Physical Status System (ASA PS-anesthesiologic screening). The main outcome measures were the relationship between perioperative laboratory results, intraoperative surgical parameters and the incidence of immediate postoperative in-hospital complications with the preoperative frailty status.

**Results:**

116 consecutive women 60 years and older (BMI 24.8 ± 5.2 kg/m^2^) with OC, who underwent elective oncological surgery in University Medical Center Mainz between 2008 and 2019 were preoperatively classified with the selected global health assessment tools as frail or non-frail. The rate of preoperative anemia (hemoglobin ≤ 12 g/dl) and perioperative transfusions were significantly higher in the G8-frail group (65.9% vs. 34.1%; *p* = 0.006 and 62.7% vs. 41.8%, *p* = 0.031; respectively). In addition, patients preoperatively classified as G8-frail exhibited significantly more postoperative clinical in-hospital complications (27.8% vs. 12.5%, *p* = 0.045) independent of chronological age and BMI. In contrast, ECOG PS and ASA PS did not predict the rates of postoperative complications (all *p* values > 0.05). After propensity score matching, the complication rate in the G8-frail cohort was approximately 1.7 times more common than in the G8-non-frail cohort.

**Conclusion:**

Preoperative frailty assessment with the G8 Score identified elderly women with OC recording a significantly higher rate of postoperative in-hospital complications. In G8-frail patients, preoperative anemia and perioperative transfusions were significantly more recorded, regardless of chronological age, abnormal BMI and surgical complexity. Standardized preoperative frailty assessment should be added to clinical routine care to enhance risk stratification in older cancer individuals for surgical patient-centered decision-making.

## What does this study add to the clinical work

**Table:** 

“Influence of interdisciplinary frailty screening on perioperative complication rates in elderly ovarian cancer patients – results of a retrospective observational study.” Is the consciousness, that the preoperative frailty evaluation in women with ovarian cancer seems to be useful to standardizedly enhance the individual risk stratification of in-hospital complications.	

## Introduction

Ovarian cancer (OC) is the second most common gynaecologic malignancy in developed countries after uterine carcinomas [1]. 313,959 women were newly diagnosed in 2020 and 207,252 patients died of OC worldwide according to the Global Cancer Statistics [2]. The median age of OC patients is 68 years (ys) and 43% of OC cases, as well as 64% of deaths from OC occurred in women over 65ys of age [3]. Standardized treatment requires complete surgical staging by laparotomy feasible with multi-visceral resections to reduce the postoperative tumor burden as radically as possible [4].

Increased chronological age is associated with more aggressive and advanced disease stages requiring adequately multimodal treatment options [5], but the independent role of higher age on morbidity and mortality remains controversial discussed [6, 7]. In addition, the elderly population is less likely to undergo all types of standardized multi-dimensionally oncological investigations resulting in an overall poorer outcome in older cancer patients [8–10]. Equally, the prognostic influence of an abnormal BMI on the postoperative outcomes remains unclear [11]. The obese-paradox can be explained by the fact that on the one hand a higher BMI constitutes a risk factor for tumor developing as well as for increasing surgical complexity with higher incidences of wound infections, more intraoperative blood loss and longer operating times [12, 13]. Otherwise underweighting is also a prognostic parameter for shorter postoperative survival [13].

Frailty, defined as a multidimensional aging-related clinical syndrome of decreased homeostatic reserves and function due to multiple organ systems and characterized by vulnerability to adverse health outcomes [14], is generally more prevalent in the elderly population and particularly higher in female cancer patients [15, 16]. Overall, the chronically underdiagnosed condition “frailty” reaches a prevalence ranging between 11 and 43% in the general population [17]. The need for preoperative frailty status detection is justified by the fact that surgical interventions are highly stressful and the frail cohort is less able to tolerate and adapt to radical procedures [18, 19]. This relationship between surgical distress and impaired frailty status especially affects OC patients [10]. At time of diagnoses, most advanced diseases requiring an impaired global health status based on large quantities of intra-abdominal ascites and greater tumor burden were examined, so that extended cytoreductive procedures should be required to reach complete macroscopic absence from tumor, as one of the strongest prognostic risk factor [20]. Therefore, it is important to provide a thorough inter-professional preoperative workup including, for example, pre-habilitation, nutrition counseling, physiotherapy, and postoperative rehabilitation in the elderly patient population [21].

To identify frail patients who might benefit from a (CGA), there are many different, screening tools to assess the individual global health status before major surgery through inconsistent and not standardized [20, 22, 23]. The G8 geriatric screening tool (G8 Score) proved to be a reproducible and particularly user-friendly screening instrument, especially in surgical disciplines, with its main focus on nutrition, mobility and comorbidities in combination with chronological age [24–26]. One main advantage of the G8 Score is, that the scoring system is collected by a simple multiple-choice questionnaire, which can be routinely administered by oncologists [27]. Furthermore, the diagnostic value of the G8 Score was validated especially for cancer patients with surgery indication to identify impairments in the CGA [28]. The Eastern Cooperative Oncology Group performance status (ECOG PS) appraises the basic functional and mental status and is commonly used in oncology disciplines [29]. The American Society of Anesthesiologists Physical Status System (ASA PS) is an internationally standardized, subjective grading system used by anesthesiologists to classify the preoperative overall medical health status of adult patients [30].

The aim of this study was to examine the relationship between the interdisciplinary examined preoperative frailty status and postoperative in-hospital complications. Moreover, we examined the association between frailty status and perioperative laboratory parameters as well as transfusion rates in a consecutive cohort of elderly OC patients undergoing standardized surgical treatment. This retrospective study should be viewed as preliminary work for subsequent prospective studies to determine whether participants benefit from preoperative routine frailty risk stratification and in the case of frailty-detection from a subsequent CGA.

## Materials and methods

This retrospective cohort analysis reports data from women with all stages of OC older than 60ys of age, treated at the University Medical Center Mainz–Johannes–Gutenberg University, Mainz, Germany, between January 2008 and December 2019. Standardized tumor de-bulking operations, primary or interval surgery after neoadjuvant chemotherapy, depending on tumor stage and the expected chance of macroscopic complete resection, were required. As part of the routine pre-surgical patient evaluation, the patients’ preoperative global health status was assessed by different assessment tools (Table 1).

**Table 1:** Global health assessment tools with frailty definitions

Global health assessment tool	Assessment definition		Frequencies *n* (%)	
G8 geriatric screening tool (*n* = 110) (**G8 Score**)	**G8-frail**: ≤ 14 points		G8-frail:	56 (50.9)
	**G8-non-frail**: > 14 points		G8-non-frail	54 (49.1)
Eastern Cooperative Oncology Group (**ECOG PS**) performance status (*n* = 100)	**ECOG 0:** Fully active, no performance restrictions		ECOG 0	28 (28.0)
	**ECOG 1:** Strenuous physical activity restricted; fully ambulatory and able to carry out light work		ECOG 1	49 (49.0)
	**ECOG 2:** Capable of all self-care but unable to carry out any work activities. Up and about > 50% of walking hours		ECOG 2	18 (18.0)
	**ECOG 3:** Capable of only limited self-care; confined to bed or chair > 50% of walking hours		ECOG 3	5 (5.0)
	**ECOG 4:** Completely disabled; cannot carry out any self-care; totally confined to bed or chair		ECOG 0	28 (28.0)
American Society of Anesthesiologists Physical Status System (**ASA PS**) (*n* = 109)	**ASA 1:** A normal healthy patient		ASA 2	48 (44.0)
	**ASA 2:** A patient with mild systemic disease		ASA 3	61 (56.0)
	**ASA 3:** A patient with severe systemic disease that limits activity but is not incapacitating			
	**ASA 4**: A patient with an incapacitating severe systemic disease that is a constant threat to life			
Age (*n* = 116)	Mean age [years] ± SD	70.88 ± 5.93	60–65 years	22 (19.0)
			65–70 years	32 (27.6)
			71–80 years	62 (53.4)
BMI (*n* = 113)	Median BMI [kg/m^2^]	25.36 [16.0–55.0]	≤ 25	61 (54.0)
			> 25	52 (46.0)

Assessment and frailty definitions utilized in the analysis

*G8 Score* G8 geriatric screening tool, *ECOG PS *eastern cooperative oncology group performance status, *ASA PS* American society of anesthesiologists physical status system, *ys* years, *n* = number of patients

Bold written words: analyzed main categories

We searched the hospital database to capture general patient characteristics (chronological age, BMI, preoperative frailty evaluation), including tumor characteristics (tumor stage (FIGO), histological grade and type), surgical parameters (postoperative residual tumor burden, surgical complexity score (SCS) and operative revisions), and postoperative events. The Body Mass Index [BMI (kg/m^2^)] was used, according to the recommendation of the World Health Organization (WHO), as the measure to classify underweight, overweight and obese in adults [31]. The rates of perioperative clinical and surgical complications were systematically derived according to the International Statistical Classification of Diseases and Related Health Problems (ICD-10) [32] corresponding to the Veteran Affairs’ National Surgical Quality Improvement Program (NSQIP) [33, 34]. The automated recording of postoperative complications through systematic coding implies the completeness and reproducibility of the documentation.

To retrospectively characterize the surgical effort, we used the reported postoperative tumor burden in the operation report and the surgical complexity score (SCS) by Aletti et al. [35]. SCS describes the level of difficulty and extent of surgical cytoreduction in patients with advanced OC and is divided into low, intermediate, and high complexity score groups [21]. Operative revisions were defined as the indication of a re-operative procedure caused by suture or fascia dehiscences, potentially secondary wound closure needed, mechanical ileus or insufficiencies of bowel anastomoses as well as secondary hemorrhage.

Long-term follow-up data were collected through telephone calls, written inquiries to the patients or their physicians, and by checking the available patient clinical records up to February 2021.

The *G8 geriatric screening tool* (G8 Score), established in 2011 by Bellera et al. is a geriatric screening tool, recommended by the Société Internationale d’Oncologie Gériatrique (SIOG) [25] and was calculated routinely by oncologists or geriatricians. The G8 Score consists of seven questions from the Mini Nutritional Assessment (MNA) questionnaire and includes chronological age, as the 8th item in its calculation [36]. The scoring system ranges from zero points (heavily impaired-G8-frail) to seventeen points (not at all impaired – G8-non-frail) with the original cut-off value of ≤ 14 points as an indication of frailty [37].

The *Eastern Cooperative Oncology Group performance status* (ECOG PS), as one of the most common methods to measure physiological reserves and functional status in cancer patients, was assigned individually by the operating surgeon or the attending oncologists during the pre-operative consultation. The degree of functional impairment is divided into six categories, as a simplification of 1948 first described Karnofsky status [38]. Five points, the maximum of the score, represent clinical death, while a value of zero points represents normal unrestricted daily live activities before disease [39].

The *American Society of Anesthesiologists Physical Status System* (ASA PS) is an internationally commonly used subjective rating system, to categorize the preoperative overall medical health status of adult patients, first described in 1941 [40]. The current ASA PS classification was proposed in 1963 by Dripps et al. [41]. The classification instrument ranges from ASA 1 – a healthy patient without any systemic disturbance to ASA 6 – a brain-dead patient whose organs are removed for organ donation [30, 42]. According to the inclusion criteria of the current study, emergency surgeries were excluded, thus ASA Class 5 and 6 were not involved in the evaluation.

Statistical analyses were performed with SPSS statistical software program, version 23.0 V5 R (SPSS Inc, Chicago, IL, U.S.A.) and StatalC 16 V5. Patients’ characteristics were given in absolute and relative frequencies (categorical data) as mean (± standard deviation (SD)) for normally distributed or as median with their interquartile ranges (IQR) for non-parametric data. The frequency of distribution of categorical variables was compared with Fisher’s exact test and correlations were calculated with Pearson correlation. First, preoperative frailty screening was performed according to the current categorization of the three examined global health assessment tools to detect the preoperative frailty status of the OC patients (Table 1). Moreover, to assess the usability in a clinical context, we divided the study cohort into three groups, according to the chronological age (60–65 ys vs. 65–70 ys vs. 71–80 ys), as well as two groups with lower and higher BMI than the median. Second, we investigated the impact of these various patient evaluation systems on perioperative complications using the exact chi-square test. A two-tailed *p* value < 0.05 was considered statistically significant. All tests should be understood as explorative analysis; no adjustment for multiple testing has been made.

To eliminate the concurrent effects of BMI and chronological age as other possibly predictive factors of the preoperative frailty as baseline confounder, these variables we determined the propensity score model (PSM).

## Results

**Fig. 1:**
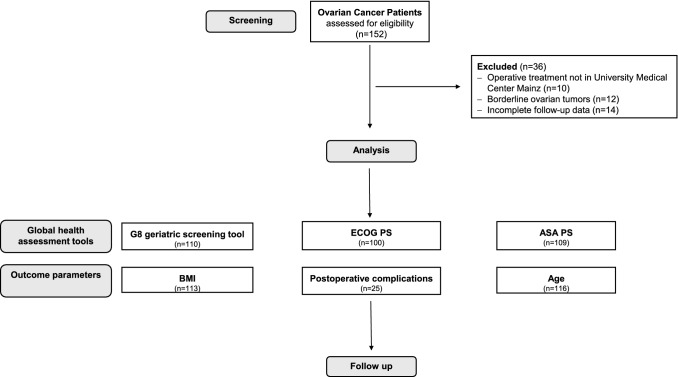
Consort statement

A total of 116 women aged 60 ys and older (mean 70.9 ± 5.9 ys with a mean BMI 24.8 ± 5.2 kg/m^2^) were eligible for this study (for details see Fig. 1). Patients’ characteristics are presented in Table 2. The preoperative global health status was determined for all study participants by the three interdisciplinary global health assessment tools, according to the international determined classification definitions (Table 1).

**Table 2:** Patient characteristics ovarian cancer (*n* = 116)

Parameter	Frequencies mean ± SD, median [25–75%]
**Mean chronological age** [years]	70.9 ± 5.9
**Mean Body Mass Index** [kg/m^2^]	24.8 ± 5.2
**Mean length of hospitalization** [days]	15.97 ± 11.82
**Median follow-up time** [months]	26.0 [12.0–39.0]
**Tumor stage (FIGO)** number of patients (%)	
** I**	13 (11.2)
Ia	8 (6.9)
Ib	1 (0.9)
Ic	4 (3.4)
** II**	6 (5.2)
IIa	2 (1.7)
IIb	4 (3.4)
** III**	74 (63.8)
IIIa1i + IIIa1ii + IIIa2	2 + 3 + 0 (1.7 + 2.6 + 0.0)
IIIb	13 (11.2)
IIIc	56 (48.3)
**IV**	17 (14.7)
Iva	2 (1.7)
IVb	15 (12.9)
**Early ovarian cancer**	
(FIGO I–FIGO IIA)	15 (12.9)
**Advanced ovarian cancer**	
(FIGO > IIA)	97 (83.6)
**Histological grade**	
G1	6 (5.3)
G2	21 (18.4)
G3	87 (76.3)
**Histological type**	
Serous	86 (74.1)
Low grade	5 (4.3)
High grade	81 (69.8)
Others	
(e.g., Mucinous, endometrioid, others, unknown)	30 (25.7)
**Postoperative residual tumor burden**	
None	67 (58.3)
Present	46 (40.0)
Unknown	2 (1.7)
**SCS–Surgical complexity score**	
Low	38 (33.3)
Intermediate	55 (48.2)
High	21 (18.4)
**Events**	
Relapse	49 (42.2)
Death due to OC	47 (40.5)
Death	55 (47.4)
Death within 60 days	4 (3.4)
**Postoperative in-hospital complications**	
Total	25 (21.6)
Cardial	7 (6.0)
Pulmonary	6 (5.2)
Nephrological	1 (0.9)
Thromboembolic	1 (0.9)
Multiorgan failure	8 (6.9)
Death within hospital stay	2 (1.7)

*SD *standard deviation, *FIGO *International Fédération of Gynecology and Obstetrics, *OC* ovarian cancer, *G* histological grade, *SCS *surgical complexity score, *n* = number of patients

Bold written words: analyzed main categories

**Fig. 2:**
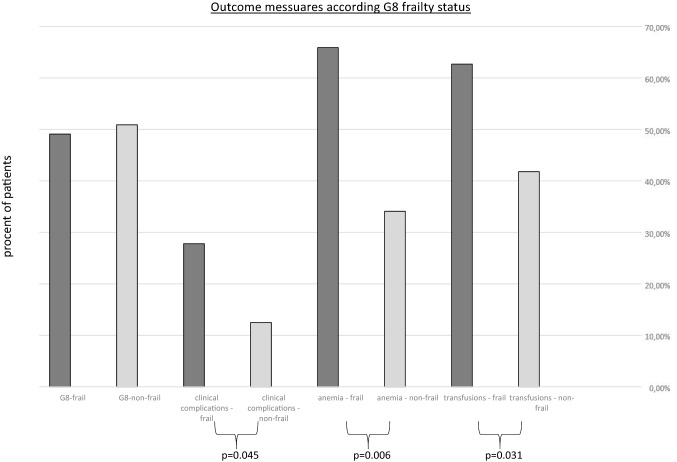
Outcome measures according G8 frailty status

Patients preoperatively classified as G8-frail were significantly more likely to have in-hospital complications, such as nosocomial pneumonia, cardiac failure and thrombosis or pulmonary embolism, as well as acute renal failure (total complications: G8-frail: 27.8% vs. G8-non-frail: 12.5%, *p* = 0.045) (Table 3). In contrast, postoperative clinical complications were not associated with ECOG PS and ASA PS nor with chronological age or higher BMI in the cohort. Overall, from a surgical perspective, no differences in surgical radicality were found depending to the preoperative frailty status (*p* values of SCS all > 0.05). Furthermore, rates of operative revisions correlate significantly with higher chronological age (age 60–65 ys: 0.0% vs. age > 65–70 ys: 23.8% and age > 70 ys: 76.2%, *p* = 0.024; respectively). Nevertheless, preoperative evaluation as G8-frail should raise the awareness that an increased revision rate occurs in everyday clinical practice (G8-frail: 25.9% vs. G8-non-frail: 12.5%, *p* = 0.073). In addition, preoperative classification as “frail” using the G8 Score was significantly associated with preoperative anemia (G8-frail: 65.9% vs. G8-non-frail: 38.5%, *p* = 0.006) and perioperative transfusion rates (G8-frail: 62.7% vs. G8-non-frail: 41.8%, *p* = 0.031) (Table 3 and Fig. 2). In contrast, the conventionally used global health assessment tools (ECOG PS and ASA PS) were not associated with preoperative anemia or transfusion indication.

**Table 3:** Surgical characteristics in correlation with frailty status

	Clinical complications	Anemia [hemoglobin g/dl]		Transfusions	Surgical complexity score (SCS)			Operative revisions	Length of hospital stay ≥ 16 days	Death within 60 days
		< 12	> 12		1	2	3			
G8-non-frail	7 (12.5)	14 (34.1)	40 (64.5)	23 (41.8)	18 (48.6)	26 (51.0)	12 (57.1)	7 (12.5)	16 (28.6)	1 (7.1)
G8 frail	15 (27.8)	27 (65.9)	25 (38.5)	32 (62.7)	19 (51.4)	25 (49.0)	9 (42.9)	14 (25.9)	23 (42.6)	3 (13.6)
***p* value G8 Score**	**0.045**	**0.006**		**0.031**	0.822			*0.073*	0.124	0.546
ECOG 0	4 (14.3)	10 (26.3)	17 (27.9)	13 (46.4)	8 (22.9)	14 (30.4)	6 (31.6)	2 (7.1)	9 (32.1)	0 (0.0)
ECOG 1	10 (20.4)	17 (44.7)	32 (52.5)	19 (39.6)	21 (60.0)	20 (43.5)	8 (42.1)	12 (24.5)	16 (32.7)	2 (11.8)
ECOG 2	4 (22.2)	8 (21.1)	10 (16.4)	13 (72.2)	5 (14.3)	9 (19.6)	4 (21.1)	3 (16.7)	10 (55.6)	1 (11.1)
ECOG 3	3 (60.0)	3 (7.9)	2 (3.3)	2 (50.0)	1 (2.9)	3 (6.5)	1 (5.3)	2 (40.0)	4 (80.0)	0 (0.0)
***p* value ECOG PS**	0.147	0.666		0.131	0.832			0.171	*0.072*	0.797
ASA 2	8 (16.7)	13 (27.7)	27 (45.8)	20 (41.7)	19 (51.4)	19 (35.8)	10 (52.6)	6 (12.5)	12 (25.0)	2 (20.0)
ASA 3	14 (23.0)	34 (72.3)	32 (54.2)	36 (59.0)	18 (48.6)	34 (64.2)	9 (47.4)	13 (21.3)	27 (44.3)	2 (7.4)
***p* value ASA PS**	0.417	*0.056*		*0.072*	0.245			0.229	**0.037**	0.273
Age 60–65 years	4 (17.4)	6 (14.3)	13 (19.1)	9 (15.8)	9 (23.7)	8 (14.5)	4 (19.0)	0 (0.0)	4 (9.8)	0 (0.0)
Age > 65—70 years	3 (13.0)	8 (19.0)	21 (30.9)	10 (17.5)	6 (15.8)	19 (34.5)	6 (28.6)	5 (23.8)	11 (26.8)	0 (0.0)
Age > 70 years	16 (69.6)	28 (66.7)	34 (50.0)	38 (66.7)	23 (60.5)	29 (50.9)	11 (52.4)	16 (76.2)	26 (63.4)	4 (16.7)
***p* value age**	0.163	0.222		**0.028**	0.361			**0.024**	0.133	0.297
BMI < 25 kg/m^2^	10 (18.5)	27 (64.3)	30 (45.5)	33 (60.0)	22 (38.6)	25 (43.9)	10 (17.5)	9 (15.5)	20 (34.5)	2 (11.1)
BMI ≥ 25 kg/m^2^	11 (19.3)	15 (35.7)	36 (54.5)	24 (45.3)	14 (25.5)	30 (54.5)	11 (20.0)	12 (21.8)	21 (38.2)	2 (11.1)
***p* value BMI**	0.917	*0.056*		0.126	0.325			0.389	0.683	1.00

Assessment and frailty definitions utilized in the analysis

*G8 Score* G8 geriatric screening tool, *ECOG PS* Eastern Cooperative Oncology Group performance status, *ASA PS* American society

of anesthesiologists physical status system, *ys* years, *n* = number of patients

Bold written words: analyzed main categories, bold written numbers: significant results (*p* < 0.05), italic written numbers:* p* < 0.1

In PSM, chronological age and BMI did not persist as statistically significant prognostic factors for complications in the matched groups, independent of G8 Score, because the accumulation of complications did not correlate with age and BMI. The complication rate in the G8-frail cohort was 1.7 times more common than in the G8-non-frail cohort.

## Discussion

This study examines the prognostic influence of various interdisciplinary frailty assessment tools on the prevalence of postoperative in-hospital complications, anemia and transfusion rates in OC patients. We assessed the frailty status with three interdisciplinary global health assessment tools: G8 Score, ECOG PS and ASA PS and validated their prognostic impact in a highly selected patient cohort with exclusively elderly women with OC.

Up till now, no evidence-based data examining the predictive influence of preoperative diagnosed frailty status with G8 Score for postsurgical outcomes in OC patients exist. In our study, frailty evaluation with the G8 Score proved to be the best indicator for postoperative in-hospital complications. In addition, our study group demonstrated, that G8-frail women (50.9%) suffered more often from preoperative anemia (65.9% vs. 34.1%; *p* = 0.006) and received significantly more perioperative transfusions (62.7% vs. 41.8%; *p* = 0.031). Similar results have been found by several smaller investigations, prospective and retrospective, which used a variety of frailty screening tools to examine an association between frailty and postoperative outcomes in specific surgical populations [43–45].

According to the most popular definition of “the phenotype of frailty” by Fried and colleagues, its impact on several outcome measures in oncologic surgery was validated by different study groups [46]. In the context of Clavien IV/V complications after gynecological cancer surgery, Uppal and colleagues published results of in total 6551 patients [45]. They calculated a modified frailty index with 11 variables as a predictive parameter of the need for critical care support and an increased 30-day postoperative mortality. Moreover, Inci and colleagues recently published in their prospective cohort trial with 226 women (median age 59ys, range from 18 to 87 ys) with several kinds of gynecological cancer surgery, that Fried Frailty Score could not significantly predict postoperative complications (OR: 2.41, 95%- CI [0.91–6.41], *p* = 0.077) [20]. Contrastingly to our findings, they postulated, that ECOG PS, BMI and albumin levels < 3.6 g/dl estimated severe postoperative complications. Possibly, our contrasting results might be explained by the fact that our study cohort was highly selected with executively OC patients all 60 ys of age and older (mean age: 70.9 ± 5.9 ys), almost 49.1% G8-frail women. Overall, our study group recently demonstrated the worse impact of G8-frail status on progression-free survival for OC patients [21].

The BMI as independent prognostic parameter in oncologic patients treating surgically is controversially discussed in the literature currently available [47]. Women with OC fully reflected the obesity-paradox. On the one hand, the oncological outcome of obese women is significantly worse and a higher BMI is a risk factor for developing various subtypes of ovarian tumors [11, 48, 49]. Additionally, obesity was demonstrated as a significant risk factor for developing postoperative wound infections, reporting more surgical blood loss and longer operating time, based on the increased surgical complexity [12, 13]. Otherwise, the underweight patients, almost women with advanced staged of OC with intra-abdominal ascites and greater tumor burden were most at risk of major postoperative complications, including long-term mortality (e.g., pneumonia, thromboembolic events, cardiovascular and cerebrovascular events, ICU admission or readmission) [13]. In our study, neither underweight nor overweight were independently associated with the rate of postoperative complications (all *p* values > 0.05). We explained this lack of connection with the highly association between the occurrence of postoperative complications and preoperative frailty, detecting with selected frailty assessment tools in patients with OC.

Concerning the fact that elderly, almost frail women with OC often were undertreated, Dion and colleagues examined 1119 women, of which 147 were 75 ys and older, surgically treated between 2007 and 2015 in a retrospective multicenter trial [50]. Their results showed, that surgeons modified their operative approach in elderly women. They underwent fewer bowel resections (32% vs. 67%, *p* < 0.001) and in this context the study group experienced fewer postoperative complications (22.6% vs. 38.9%, *p* < 0.001) in the sub-cohort of the elderly with an overall worse prognosis. Consequently, the qualification of preoperative frailty, especially in patients with newly diagnosed advanced OC might be useful and predictive diagnostic parameter for clinical decision-making [51, 52]. We tried to overcome this bias of different surgical effort according to the frailty status or chronological age with no differences according to the surgical complexity score. In our study cohort, operative revisions were not predicted by preoperative frailty status, in contrast to a significant correlation to increasing age (76.2% vs. 23.8% vs. 0.0%; *p* = 0.024).

However, there is no universally accepted definition of frailty that has been operationally studied with a variety of screening tools, although there is a consensus that frailty is a chronically biological state or syndrome of diminished resilience to stressors resulting from deterioration in multiple physiological systems [53–56]. Frailty encompasses a constellation of clinical attributes, including low activity levels rather than loss of skeletal muscle mass and poor endurance in combination with comorbidities and polypharmacy [14].

The main focus on nutrition, mobility and comorbidities combined with chronological age seemed to account for the strong association between preoperative frailty and the mentioned outcome parameters in the patient cohort of older women with OC [21]. This prompted our decision to examine the G8 Score as the preoperative frailty screening tool for OC patients before major de-bulking surgery. The G8 Score proved to be a practical and time-saving screening test, especially for elderly cancer patients before major abdominal surgical intervention and for identifying frail patients who could benefit from a subsequent detailed CGA [25, 57]. In contrast to the G8 Score, neither the ECOG PS, which was the standard of care used by oncologists nor the anesthesiologists’ classification system (ASA PS) could predict outcomes. Regarding an unselective cohort of women with gynecologic cancers, a German study group from the Charité University Center Berlin published the RISC-Gyn Trial, in which postoperative complications were predicted by an ECOG PS > 1 and various domains of quality of life as well as the preoperative nutrition status in 2020 [58]. Giannica and colleagues suggested that oncological surgery in elderly women with gynecological malignancies older than 70 ys with ASA PS 3 and 4 resulted in a higher rate of serve morbidity, whereas acceptable perioperative morbidity with similar median postoperative hospital stay (8 days) and mortality rate (median 3%) was recorded in total [59]. The contrasting results might be explained by our highly selected study population of executively OC patients solely with laparotomies and the fact, that the G8 Score enabled a multidimensional view of the cancer patient’s global health domains [60]. Moreover, G8 Score dichotomizes the study cohort into frail or non-frail, while ASA PS and the ECOG PC classification systems standardly range from 1 to 4.

Several limitations of our study should be noted. First, we declared analyses of a retrospective and single-institution-designed study. This may be particularly important regarding incomplete follow-ups, which was successfully reduced to a minimum of 14 patients (9.2%) by reaching out to patients and physicians through various communication channels and a comprehensive review of clinical records. Frailty status screening was offered to all women 60 ys and older with OC seen at preoperative consultation. We further sought to reduce the possible systemic errors resulting from the wide range of different surgical interventions and surgeons by operating on all patients in one University hospital according to the current national guidelines. Lastly, further studies are required to determine specific risk factors in patients undergoing oncological surgery and to specify the impact of preoperative frailty status. Large-scale projects were needed to develop protentional preoperative interventions that may limit the postoperative complication rate in the frail elderly cohort.

In conclusion, the preoperative classification as G8-frail has been shown to be a significant risk factor for increased in-hospital morbidity in elderly women with gynecological cancer [61]. Moreover, we could demonstrate the G8 Score as “an independent prognostic marker in elderly patients with OC independently of maximal surgical effort” [21]. Our findings underline the need for a standardized frailty status-detection algorithm using validated screening tools to identify the subgroup of patients, who may benefit from the full CGA to define the patients being at risk for perioperative complications. Even if it seems reasonable to establish multidisciplinary pre-habilitation programs, to identify non-oncologic issues promoting perioperative complications, to improve global health status, and especially in the subgroup of frail patients to reduce perioperative complications. Its impact goes beyond the scope of this study and might be addressed in further prospective clinical trials. In contrast, the majority of the patients were classified as non-frail and should receive standardized, maximal surgical efforts to avoid under-treatment. Consecutively, in a recently published trial from our study group, we could demonstrate the overall worse oncological outcome of patients with residual tumor burden in accordance to the preoperative frailty-stratification (G8-frail, no-residual tumor burden: vs. G8-non-frail, non-residual tumor burden) [21]. In the subgroup of frail patients, an individualized patient-centered treatment option must be discussed. Further prospective research should be initiated to confirm these findings and to improve the identification of the preoperatively frail patient who requires individualized modified therapy management in a standardized manner.

## Data Availability

All data generated or analyzed during this study are included in this article and its supplementary material files. Further enquiries can be directed to the corresponding author.

## Abbreviations

ASA PS: American Society of Anesthesiologists Physical Status System
BMI: Body Mass Index
CGA: Comprehensive geriatric assessment
CI: Confidence interval
ECOG PS: Eastern Cooperative Oncology Group performance status
FIGO: Fédération Internationale de Gynécologie et d'Obstétrique
G8 Score: G8 geriatric screening tool
IQR: Interquartile ranges
MNA: Mini-nutritional assessment
OC: Ovarian cancer
OR: Odds ratio
SCS: Surgical complexity score
SIOG: Société Internationale d’Oncologie Gériatrique
SD: Standard deviation
WHO: World Health Organization
YS: Years

## References

[1] Martins FC (2020). Clinical and pathological associations of PTEN expression in ovarian cancer: a multicentre study from the ovarian tumour tissue analysis consortium. Br J Cancer.

[2] Sung H (2021). Global cancer statistics 2020: GLOBOCAN estimates of incidence and mortality worldwide for 36 cancers in 185 countries. CA Cancer J Clin.

[3] Courtney-Brooks M (2012). Frailty: an outcome predictor for elderly gynecologic oncology patients. Gynecol Oncol.

[4] Hengeveld E, Zusterzeel P, Lajer H, Høgdall C, Rosendahl M (2019). The value of surgical staging in patients with apparent early stage epithelial ovarian carcinoma. Gynecol Oncol.

[5] Gibson SJ, Fleming GF, Temkin SM, Chase DM (2016). The application and outcome of standard of care treatment in elderly women with ovarian cancer: a literature review over the last 10 years. Front Oncol.

[6] Quaglia A (2009). The cancer survival gap between elderly and middle-aged patients in Europe is widening. Eur J Cancer.

[7] Kumar A (2017). Functional not chronologic age: frailty index predicts outcomes in advanced ovarian cancer. Gynecol Oncol.

[8] Rauh-Hain JA (2015). Management for elderly women with advanced-stage, high-grade endometrial cancer. Obstet Gynecol.

[9] Wimberger P (2006). Impact of age on outcome in patients with advanced ovarian cancer treated within a prospectively randomized phase III study of the arbeitsgemeinschaft gynaekologische onkologie ovarian cancer study group (AGO-OVAR). Gynecol Oncol.

[10] Yao T, DeJong SR, McGree ME, Weaver AL, Cliby WA, Kumar A (2019). Frailty in ovarian cancer identified the need for increased postoperative care requirements following cytoreductive surgery. Gynecol Oncol.

[11] Foong KW, Bolton H (2017). Obesity and ovarian cancer risk: a systematic review. Post reproductive health.

[12] Ri M, Aikou S, Seto Y (2018). Obesity as a surgical risk factor. Anna Gastroenterol Surg.

[13] Tjeertes EE, Hoeks SS, Beks SS, Valentijn T, Hoofwijk AA, Stolker RJR (2015). Obesity–a risk factor for postoperative complications in general surgery?. BMC Anesthesiol.

[14] Fried LP (2001). Frailty in older adults: evidence for a phenotype. J GerontoMel dical Sciences.

[15] Rodríguez-Mañas L (2013). Searching for an operational definition of frailty: a Delphi method based consensus statement. The frailty operative definition-consensus conference project. J Gerontol Series A.

[16] Handforth C (2015). The prevalence and outcomes of frailty in older cancer patients: a systematic review. Ann Oncol.

[17] Collard RM, Boter H, Schoevers RA, Oude Voshaar RC (2012). Prevalence of frailty in community-dwelling older persons: a systematic review. J Am Geriatr Soc.

[18] Dumas L, Lidington E, Appadu L, Jupp P, Husson O, Banerjee S (2021). Exploring older women’s attitudes to and experience of treatment for advanced ovarian cancer: a qualitative phenomenological study. Cancers.

[19] Anic K (2022). Impact of perioperative red blood cell transfusion, anemia of cancer and global health status on the prognosis of elderly patients with endometrial and ovarian cancer. Front Oncol.

[20] Inci MG (2021). Frailty Index for prediction of surgical outcome in ovarian cancer: results of a prospective study. Gynecol Oncol.

[21] Anic K (2021). G-8 geriatric screening tool independently predicts progression-free survival in older ovarian cancer patients irrespective of maximal surgical effort: results of a retrospective cohort study. Gerontology.

[22] Li Y (2018). Impact of frailty on outcomes after discharge in older surgical patients: a prospective cohort study. CMAJ.

[23] Sia TY, Wen T, Cham S, Friedman AM, Wright JD (2020). Effect of frailty on postoperative readmissions and cost of care for ovarian cancer. Gynecol Oncol.

[24] Revenig LM (2014). A prospective study examining the association between preoperative frailty and postoperative complications in patients undergoing minimally invasive surgery. J Endourol.

[25] Bellera C (2012). Screening older cancer patients: first evaluation of the G-8 geriatric screening tool. Ann Oncol.

[26] Bongue B, Buisson A, Dupre C, Beland F, Gonthier R, Crawford-Achour É (2017). Predictive performance of four frailty screening tools in community-dwelling elderly. BMC Geriatr.

[27] Soubeyran P (2011). Validation of the G8 screening tool in geriatric oncology: the ONCODAGE project. J Clin Oncol.

[28] Bruijnen CP (2021). Validation of the G8 screening tool in older patients with cancer considered for surgical treatment. J Geriatr Oncol.

[29] Repetto L (2002). Comprehensive geriatric assessment adds information to Eastern Cooperative Oncology Group performance status in elderly cancer patients: an italian group for geriatric oncology study. J Clin Oncol.

[30] Sankar A, Johnson S, Beattie W, Tait G, Wijeysundera D (2014). Reliability of the American society of anesthesiologists physical status scale in clinical practice. Br J Anaesth.

[31] Organization WH (2000). Obesity: preventing and managing the global epidemic.

[32] Organization WH (2004). The International Statistical Classification of Diseases and Health Related Problems ICD-10: Tenth Revision. Volume 1: Tabular List.

[33] Khuri SF (1998). The department of veterans affairs' NSQIP: the first national, validated, outcome-based, risk-adjusted, and peer-controlled program for the measurement and enhancement of the quality of surgical care. National VA Surgical Quality Improvement Program. Ann Surg.

[34] Robinson TN, Wu DS, Pointer L, Dunn CL, Cleveland JC, Moss M (2013). Simple frailty score predicts postoperative complications across surgical specialties. The Am J Surg.

[35] Aletti GD (2007). A new frontier for quality of care in gynecologic oncology surgery: multi-institutional assessment of short-term outcomes for ovarian cancer using a risk-adjusted model. Gynecol Oncol.

[36] Vellas B (1999). The mini nutritional assessment (MNA) and its use in grading the nutritional state of elderly patients. Nutrition.

[37] Hamaker ME, Jonker JM, de Rooij SE, Vos AG, Smorenburg CH, van Munster BC (2012). Frailty screening methods for predicting outcome of a comprehensive geriatric assessment in elderly patients with cancer: a systematic review. Lancet Oncol.

[38] Oken MM (1982). Toxicity and response criteria of the eastern cooperative oncology group. Am J Clin Oncol.

[39] Rodin MB, Mohile SG (2007). A practical approach to geriatric assessment in oncology. J Clin Oncol.

[40] Saklad M (1941). Grading of patients for surgical procedures. The J Am Soc Anesthesiol.

[41] Dripps RD, Lamont A, Eckenhoff JE (1961). The role of anesthesia in surgical mortality. JAMA.

[42] Fitz-Henry J (2011). The ASA classification and peri-operative risk. The Ann Royal Coll Surg Engl.

[43] Birkelbach O (2019). Routine frailty assessment predicts postoperative complications in elderly patients across surgical disciplines–a retrospective observational study. BMC Anesthesiol.

[44] Rothenberg KA (2019). Association of frailty and postoperative complications with unplanned readmissions after elective outpatient surgery. JAMA Netw Open.

[45] Uppal S, Igwe E, Rice LW, Spencer RJ, Rose SL (2015). Frailty index predicts severe complications in gynecologic oncology patients. Gynecol Oncol.

[46] Chambers LM (2021). Modified frailty index predicts postoperative complications in women with gynecologic cancer undergoing cytoreductive surgery and hyperthermic intraperitoneal chemotherapy. Gynecol Oncol.

[47] "S3-Leitlinie Diagnostik, Therapie und Nachsorge maligner Ovarialtumoren "

[48] Pavelka JC (2006). Effect of obesity on survival in epithelial ovarian cancer. Cancer.

[49] Olsen CM, Green AC, Whiteman DC, Sadeghi S, Kolahdooz F, Webb PM (2007). Obesity and the risk of epithelial ovarian cancer: a systematic review and meta-analysis. Eur J Cancer.

[50] Dion L (2020). Ovarian cancer in the elderly: time to move towards a more logical approach to improve prognosis—a study from the FRANCOGYN group. J Clin Med.

[51] Handley K (2022). "Frailty repels the knife: the impact of frailty index on surgical intervention and outcomes. Gynecol Oncol.

[52] Filippova OT (2021). Frailty based on the memorial Sloan Kettering Frailty Index is associated with surgical decision making, clinical trial participation, and overall survival among older women with ovarian cancer. Gynecol Oncol.

[53] Winograd CH, Gerety MB, Chung M, Goldstein MK, Dominguez F, Vallone R (1991). Screening for frailty: criteria and predictors of outcomes. J Am Geriatr Soc.

[54] Rockwood K, Fox RA, Stolee P, Robertson D, Beattie BL (1994). Frailty in elderly people: an evolving concept. CMAJ.

[55] Gill TM, Baker DI, Gottschalk M, Peduzzi PN, Allore H, Byers A (2002). A program to prevent functional decline in physically frail, elderly persons who live at home. N Engl J Med.

[56] Hogan DB, MacKnight C, Bergman H (2003). The Canadian initiative on frailty and aging. Aging Clin Exp Res.

[57] Martinez-Tapia C (2017). Prognostic value of the G8 and modified-G8 screening tools for multidimensional health problems in older patients with cancer. Eur J Cancer.

[58] Inci MG et al. (2020) Role of predictive markers for severe postoperative complications in gynecological cancer surgery: a prospective study (RISC-Gyn Trial). Int J Gynecol Cancer vol. 30 (12) 10.1136/ijgc-2020-001879 33246921

[59] Giannice R, Foti E, Poerio A, Marana E, Mancuso S, Scambia G (2004). Perioperative morbidity and mortality in elderly gynecological oncological patients (≥ 70 Years) by the American society of anesthesiologists physical status classes. Ann Surg Oncol.

[60] Velghe A, Petrovic M, De Buyser S, Demuynck R, Noens L (2014). Validation of the G8 screening tool in older patients with aggressive haematological malignancies. Eur J Oncol Nurs.

[61] Anic K (2022). Frailty assessment tools predict perioperative outcome in elderly patients with endometrial cancer better than age or BMI alone: a retrospective observational cohort study. J Cancer Res Clin Oncol.

